# Impact of senolytic treatment on immunity, aging, and disease

**DOI:** 10.3389/fragi.2023.1161799

**Published:** 2023-10-10

**Authors:** Erica C. Lorenzo, Blake L. Torrance, Laura Haynes

**Affiliations:** ^1^ UConn Health Center on Aging, University of Connecticut School of Medicine, Farmington, CT, United States; ^2^ Department of Immunology, University of Connecticut School of Medicine, Farmington, CT, United States

**Keywords:** senescence, immune cells, senolytics, immunosenescence, aging, D+Q, fisetin, infection

## Abstract

Cellular senescence has been implicated in the pathophysiology of many age-related diseases. However, it also plays an important protective role in the context of tumor suppression and wound healing. Reducing senescence burden through treatment with senolytic drugs or the use of genetically targeted models of senescent cell elimination in animals has shown positive results in the context of mitigating disease and age-associated inflammation. Despite positive, albeit heterogenous, outcomes in clinical trials, very little is known about the short-term and long-term immunological consequences of using senolytics as a treatment for age-related conditions. Further, many studies examining cellular senescence and senolytic treatment have been demonstrated in non-infectious disease models. Several recent reports suggest that senescent cell elimination may have benefits in COVID-19 and influenza resolution and disease prognosis. In this review, we discuss the current clinical trials and pre-clinical studies that are exploring the impact of senolytics on cellular immunity. We propose that while eliminating senescent cells may have an acute beneficial impact on primary immune responses, immunological memory may be negatively impacted. Closer investigation of senolytics on immune function and memory generation would provide insight as to whether senolytics could be used to enhance the aging immune system and have potential to be used as therapeutics or prophylactics in populations that are severely and disproportionately affected by infections such as the elderly and immunocompromised.

## Introduction

Cellular senescence is defined as a state of irreversible replicative arrest initiated via various cell-cycle regulating pathways following extensive extracellular and/or intracellular stress. This cellular process is indispensable for normal fetal development (Storer et al., 2013) and is important in tissue repair and wound healing (Reyes et al., 2022; Samdavid Thanapaul et al., 2022), prevention of tumorigenesis (Campisi, 2001), and in halting division of damaged cells or cells that have reached their replicative capacity (Herbig et al., 2004). Studies exploring the fundamentals and consequences of senescent cells (SCs) have revealed their highly dynamic nature encompassing both positive and negative effects on surrounding cells and tissues. Despite being in a state of permanent cell cycle arrest, SCs remain metabolically active and have altered phenotypes, which can vary depending on the cell type. Senescence remains an integral process in the maintenance of tissue homeostasis throughout life, however, accumulation of SCs with age is implicated in the pathophysiology of many age-related chronic diseases and in recent studies, has been associated with poor recovery from infectious disease. As a result, senescence has become an additional hallmark of biological aging and is at the forefront of many aging research studies.

## Senescent cell accumulation

**FIGURE 1:**
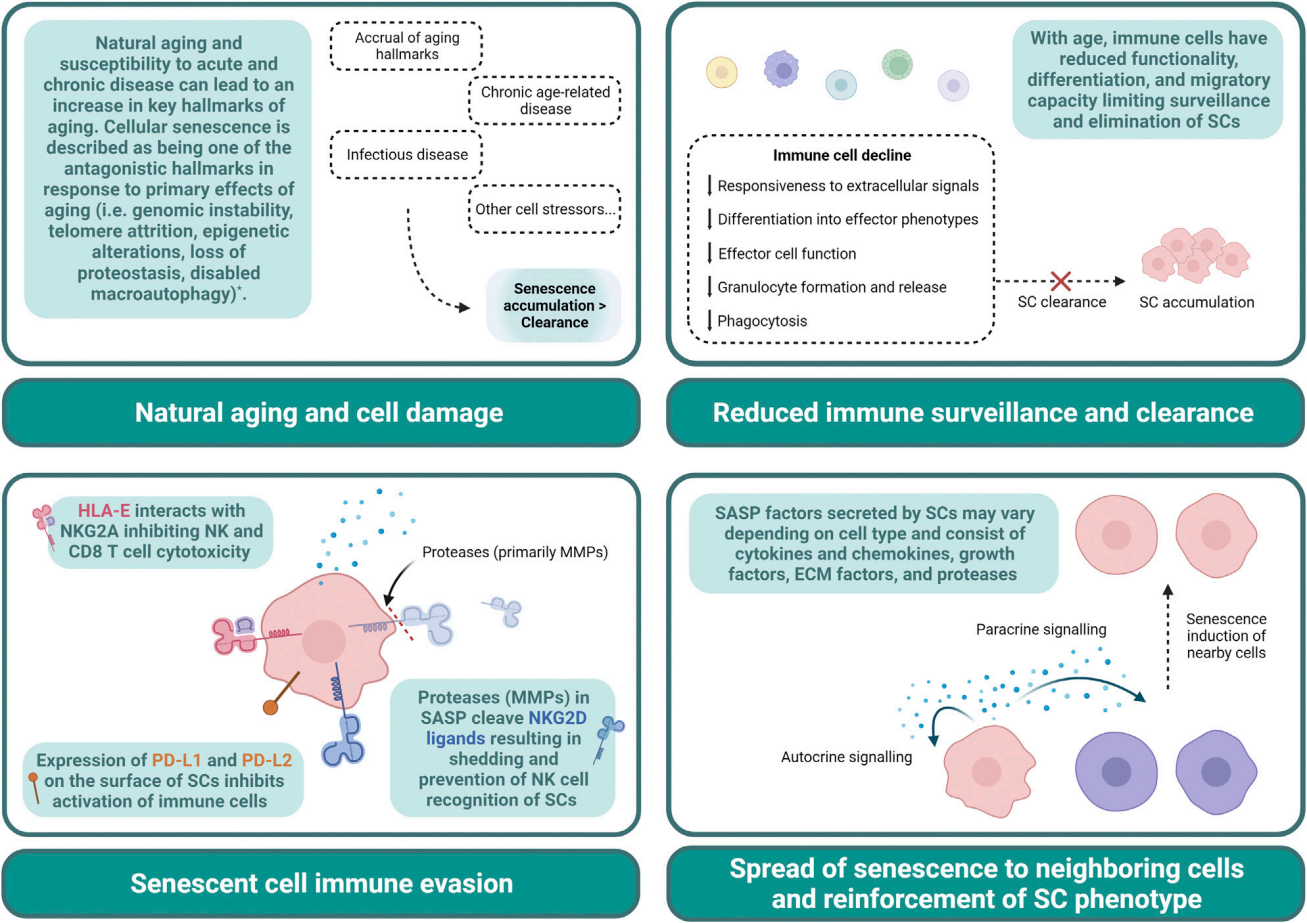
Contributing factors to senescent cell accumulation. Declines in immune cells with age result in a limited capacity to exert cytotoxic, phagocytic, and other functional properties. This can lead to an impaired clearance of SCs allowing for accumulation (**Top left panel**). Natural aging, infectious disease, chronic age-related diseases, and other cell stressors result in senescence development, and may result in an increase in SC development that outpaces removal (**Top right panel**). SCs can evade immune cell clearance by expressing HLA-E, PD-L1, PD-L2, and shed NKG2D ligands. Proteases such as MMPs secreted in SASP contribute to the cleavage of these ligands (**Bottom left panel**). SASP can reinforce SC phenotype via autocrine signaling and can induce senescence in surrounding cells via paracrine signaling. SASP contributes to cell dysfunction and the pervasive nature of SCs (**Bottom right panel**). ^*^ The hallmarks of aging have been thoroughly described in Lopez-Otin et al., 2023. ECM, extracellular matrix; HLA-E, major histocompatibility complex, class I, E; MMP, matrix metalloproteinase; NK, natural killer; NKG2A, natural killer group 2 member A; NKG2D, natural killer group 2 member D; PD-L, programmed cell death ligand; SASP, senescence-associated secretory phenotype; SC, senescent cell.

*In vitro* and *in vivo* models have led to identification of key mechanisms involved in senescence induction pathways, SC function, their biological impact, and pathogenesis. Although the SC burden across human tissues and their contribution to disease is not entirely understood, murine studies have suggested SCs represent a very small proportion of overall cells in various tissues. Transplanting relatively small numbers of SCs into young mice, for example, is sufficient to cause disease and physical dysfunction (Xu et al., 2018). Other research has demonstrated, however, there is heterogeneity between SC populations, which is dependent on cell type and method of senescence induction. As a result, it is possible that SCs with distinct phenotypes may differentially contribute to SC-related disease burden and pathology. It is also possible that in humans, disease associated SCs may represent a larger proportion of cells in the tissue than in murine models and have a higher rate of accumulation. The main factors contributing to SC accumulation are described here and summarized in Figure 1.

Natural cellular aging or response to stress and damage can trigger senescence pathways, contributing to their accumulation. Other factors include an age-related decline in immune surveillance and failure of the immune system (specifically, NK cells, T cells, and macrophages) to efficiently clear SCs (Kale et al., 2020). In healthy tissue, many of the inflammatory factors and damage signals secreted by SCs (i.e., senescence-associated secretory phenotype, SASP) will typically result in recruitment of immune cells to mediate this clearance (Sagiv and Krizhanovsky, 2013). With age and disease, however, the accumulation of SCs may surpass the rate of clearance, increasing the SC burden in tissues. With aging, in particular, declines in immune cell trafficking and clearance mechanisms further exacerbate SC accumulation and limit responsiveness to SASP signals (Ovadya et al., 2018). It has also been shown that senescent immune cells (iSCs) themselves can impact surrounding tissues. In a genetic mouse model of senescence where DNA repair gene *Ercc1* was selectively deleted in hematopoietic cells, iSCs were shown to induce senescence of other non-immune cells and increase tissue damage (Yousefzadeh et al., 2021). Moreover, SCs also develop mechanisms of immune evasion. Pro-inflammatory cytokines secreted by senescent dermal fibroblasts, for example, can lead to expression of MHC molecule HLA-E, which inhibits NK and CD8 T cell responses and clearance of SCs (Pereira et al., 2019). SCs have been demonstrated to shed NKG2D ligands evading detection by primarily NK and CD8 T cells (Munoz et al., 2019) as well as upregulate expression of PD-L1, inhibiting immune cell activation (Wang TW, et al., 2022b; Onorati et al., 2022).

## Senescent cell biomarkers

Many features of SCs have been identified as key biomarkers such as DNA damage foci, expression of cell cycle regulators p53, p16^INK4a^, or p21^Cip1^, presence of senescence-associated β-galactosidase (SA-β-gal), shortened telomeres, mitochondrial damage, altered nutrient sensing and cell signaling, all of which have now been thoroughly reviewed by others (González-Gualda et al., 2021). While the majority of animal models of senescence depend on genetic modifications in order to delete p21^Cip1^ or p16^INK4a^ expressing cells, it is clear these genes are not universally or uniformly expressed when comparing senescence across tissues. A small 2020 study examining p16 and p21 expression in various tissue samples of healthy young, middle aged, and older adults suggests there is an increase in these markers with age in specific tissues (e.g., pancreas, dermis, and kidney), but not in others (e.g., lung, cardiac, or skeletal muscle) (Idda et al., 2020). SCs can also express urokinase-type plasminogen activator receptor (uPAR), which promotes the degradation of the extracellular matrix during fibrinolysis and wound healing (Smith and Marshall, 2010). Soluble uPAR that results from proteolytic cleavage has also been identified as a SASP factor (Coppé et al., 2008). uPAR CAR-T cells have been shown eliminate SCs and SC-related pathology in mice (Amor et al., 2020). Increase in lysosomal activity and SAβ-gal expression has also been targeted as a unique drug delivery mechanism where gal-encapsulated cytotoxic drugs can selectively eliminate SCs in a model of chemotherapy-induced senescence (Munoz-Espin et al., 2018). Following endocytosis and fusion of the capsule with lysosomes the cytotoxic drugs are released into the cell allowing for drug delivery into SCs and reduction other cytotoxic effects of chemotherapeutic drugs (Munoz-Espin et al., 2018). These examples demonstrate the use of SC biomarkers to develop novel therapeutic strategies. iSCs have also been shown to express classic hallmarks of senescence and cell exhaustion; PD-1 has been identified as another potential hallmark of T cell senescence (Janelle et al., 2021).

Production of SASP is another important biomarker and feature in the establishment of the senescence phenotype. SCs exert their profound effects primarily through the secretion of senescence-associated soluble and insoluble factors and proteins which influence surrounding cells and tissues and reinforces their own SC phenotype (e.g., inflammatory cytokines and chemokines, proteases, growth factors, and matrix proteins) (Coppé et al., 2010). Termed the SASP, these factors induce senescence in neighboring cells and consequently contribute to the aged tissue environment (Acosta et al., 2013). Although our knowledge is relatively limited, we have learned through model comparisons that senescence and SASP are overall heterogenous (Coppé et al., 2010; Hernandez-Segura et al., 2017). Several core SASP factors, however, are shared irrespective of senescence induction model, cell type, and species (e.g., growth/differentiation factor-1, serine protease inhibitors, extracellular matrix proteins) (Basisty et al., 2020).

Taken together, other methods to alleviate SASP (senomorphics) and to eliminate SCs (senolytics) have been introduced to circumvent this lack of natural clearance in attempts to restore tissue homeostasis, function, and in some cases reverse disease (Chaib et al., 2022). By usage of these different methods, it is possible to enhance clearance of SCs, and improve immune and non-immune cell function, although more studies are needed to examine these hypotheses. Because immune cells play an integral role in the clearance of SCs, we focus this review on the use of senolytics and the effects on the immune system as well as their impact on the response to disease, either age-related or infectious, and considerations before therapeutic use.

## Senolytic drugs and immune cells: a broad overview

Senolytic drugs have become a valuable tool in examining the effects of senescence *in vitro* and *in vivo.* They function by inhibiting senescent cell anti-apoptotic (SCAP) and pro-survival pathways to selectively force initiation of apoptosis in SCs, leaving quiescent and proliferating cells intact (Zhu et al., 2015; Kirkland et al., 2017). There are many candidate senotherapies, but we will focus on the three that are currently in clinical trials with patients 65+ years of age with various conditions (e.g., Alzheimer’s disease and osteoarthritis): fisetin, dasatinib, and quercetin (usually taken in combination, D + Q) (Kirkland and Tchkonia, 2020). Fisetin and D + Q, in contrast to other senolytics like navitoclax, have been chosen due to their better *in vivo* senolytic activity and more favorable safety profile in clinical trials (Wilson et al., 2010; Raffaele and Vinciguerra, 2022). They also have the ability to inhibit multiple SCAP pathways simultaneously, allowing for more specific targeting of SCs, in a wider range of SC types when compared to navitoclax (Zhu et al., 2016).

Research in cancer and other models of senescence induction in young animals, *ex vivo*, or *in vitro* have expanded our understanding of the immunomodulatory effects of senolytic drugs, however, studies in aged animals and clinical trials in adults 65+ years of age or in the context of infectious disease is very sparse and will be discussed further. Although many of the studies discussed in this review did not use these drugs with the specific intention of eliminating SCs, they highlight the direct impact senolytics can have on immune cell function. This is important as we consider bringing senolytic drugs into the clinic since immune cells and immunological memory are indispensable for clearance and control of infectious disease and play a role in age-related disease pathology. It is also important to consider that in these studies treatment with these drugs have been used at various timepoints and concentrations and via different methods of drug administration, briefly described for each.

## Senolytic drugs and innate immune cells

The innate immune system plays a crucial role in the initial protection against pathogens and foreign antigens. Its key functions are multifaceted and rapid, serving as the body’s first line of defense. One of its primary functions is pathogen recognition whereby pattern recognition receptors (PRRs) respond to conserved features termed pathogen-associated molecular patterns (PAMPs) and damage-associated molecular patterns (DAMPs) triggering inflammatory responses, phagocytosis, cell death, and cascades that aid in pathogen elimination (e.g., the complement system) (Paluden et al., 2021). Innate immune cells are also important in regulating homeostatic processes and recovery from injury (Paluden et al., 2021). Moreover, the innate immune system also triggers the adaptive immune response by presenting antigens to lymphocytes, and by providing costimulatory, cytokine, and chemokine signals to reinforce adaptive cell fate and functions further strengthening overall immune defense. Studies examining various characteristics of innate immune cell function with use of fisetin, dasatinib, and quercetin will be discussed here. To better understand how these drugs will operate as senolytics, we must also learn from studies using these drugs in other contexts to determine the expected functional and phenotypic impacts.

Fisetin, a natural flavonoid found in many foods such as cucumbers and strawberries, has demonstrated a protective and anti-inflammatory effect related to innate immune cells in several models. In a model of ischemic stroke induced by transient cerebral middle artery occlusion, fisetin was injected into young mice either 20 min prior to or 180 min post-stroke (Gelderblom et al., 2012). Fisetin (50 mg/kg of body weight) protected brain tissue against ischemic reperfusion when given before or post-stroke and significantly reduced infarct size (Gelderblom et al., 2012). It prevented infiltration of macrophages, dendritic cells, and lymphocytes in ischemic brains, which also corresponded with a reduction in TNF-α production from brain-derived macrophages and microglia (Gelderblom et al., 2012). *In-vitro*, this study demonstrated a significant reduction in TNF-α levels following lipopolysaccharide (LPS) treatment in both murine and human fisetin-treated cell cultures in a dose-dependent manner. Fisetin treatment also significantly reduced cell death in mouse microglial cultures with LPS (Gelderblom et al., 2012). Taken together, fisetin effects innate immune cell types, most notably macrophage and microglial cells, by modulating their activation, ischemic infiltration, and cytokine production following stroke, a leading cause of disability and death in adults (GBD, 2019 Stroke Collaborators, 2021).

In another study, murine derived macrophages (RAW 264.7 cells) showed a significant decrease in viability with higher doses of fisetin (10 uM and 20 uM) *in vitro*, which further declined with the addition of LPS in the cell culture media (Molagoda et al., 2021). At lower doses of fisetin ranging from 0-8 uM with the addition of LPS, there was no significant change in viability, but there was a dose-dependent decrease in levels of macrophage-related inflammatory and regulatory mediators (e.g., iNOS, COX-2, nitric oxide, PGE_2_, IL-6, and TNF-α) (Molagoda et al., 2021). *In vivo*, zebrafish larvae in the presence of fisetin demonstrated protection from endotoxic shock following injection with LPS resulting in significantly reduced mortality (Molagoda et al., 2021). 18 h post-LPS injection of zebrafish larvae in the presence of fisetin revealed a dose-dependent decrease in iNOS, COX-2, IL-6, and TNF-α mRNA expression (Molagoda et al., 2021). At 24 h post-LPS injection, zebrafish larvae demonstrated a fisetin-related dose-dependent reduction in the infiltration of macrophages and neutrophils to the site of LPS injury, suggesting that fisetin attenuated their migration and inhibits innate cell LPS inflammatory responses (Molagoda et al., 2021).

Fisetin treatment was also evaluated for its effects on a murine model of allergic asthma regulation (Huang et al., 2018). Mice were treated with either 40 mg/kg body weight (low dose, LD) or 50 mg/kg body weight (high dose, HD) of fisetin over the course of 47 days and sensitized to ovalbumin (days 0 and 15) followed by a rest period and then subsequent secondary allergen exposure to ovalbumin (secondary challenge, days 31–36) (Huang et al., 2018). Treatment with HD fisetin in sensitized and challenged mice showed a significant increase in lung function and a significant reduction in airway hyperresponsiveness (AHR) and antibody production (Huang et al., 2018). Both the LD and HD fisetin treated groups showed significantly less lung tissue damage and less infiltration of macrophages, neutrophils, leukocytes, and eosinophils in bronchoalveolar lavage fluid (BAL) when compared to the control OVA-no fisetin treated group (Huang et al., 2018). Fisetin treated groups also had a significant reduction in levels of IL-1β, TNF-α, IL-18, and IL-4, in the BAL, which can all be secreted by innate immune cells (Huang et al., 2018). Co-stimulatory molecules CD80 and CD86 expressed on antigen presenting cells including dendritic cells, were significantly reduced with fisetin treatment. Treatment with fisetin also decreased MyD88/NFκ-β signaling reducing inflammation and playing a direct role in modulating allergic asthma in this model (Huang et al., 2018). Overall, there is some evidence in various models that fisetin plays a direct anti-inflammatory role in innate immune cell responses. It can reduce their migration and responsiveness to stimuli such as LPS, preserving tissue integrity and preventing mortality in zebrafish, however, it is also clear that the cytotoxicity of high doses should be taken into consideration before use.

Dasatinib (a tyrosine kinase receptor inhibitor) and quercetin (a flavonoid) are often used in combination treatment, however they exhibit immunomodulatory effects independently. In an *ex vivo* study examining human neutrophils isolated from whole blood, incubation with different concentrations of dasatinib between 0 and 1,000 nM was observed to have a dose-dependent effect on superoxide release and production of reactive oxygen species when stimulated by a variety of different immunostimulatory agents (e.g., ultra-pure LPS and Pam3CSK4) (Futosi et al., 2012). Interestingly, this study demonstrated the sensitivity of neutrophils to dasatinib treatment. Dasatinib was found to impair or completely inhibit many critical functions of neutrophils such as integrin signaling, where activation of the Syk-tyrosine kinase pathway is blocked (Futosi et al., 2012). It did not, however, impair TNF-α signaling, phagocytosis, or bacterial killing (Futosi et al., 2012). Dasatinib impaired exocytosis of granules, cell migration, and blocked immune-complex induction of neutrophil activation (Futosi et al., 2012). It impaired cell adherence and recognition of innate immune ligands such as opsonized and unopsonized zymosan, as depicted by a reduction in superoxide release following exposure to these ligands (Futosi et al., 2012). Overall, while dasatinib may be beneficial in neutrophil-mediated diseases, caution must be exercised in cases where impairing neutrophil function would be detrimental, e.g., in cases of high risk of contracting bacterial or viral infections.

Dasatinib has been FDA approved for many years to treat several types of leukemias and other treatment resistant cancers. A 2013 study included 55 adults [men and women; mean age, 53 years; range, 20–76 years] treated with dasatinib for chronic myeloid leukemia or acute lymphoblastic leukemia (Mustjoki et al., 2013). Study measurements taken between 0 and 24 h following a single 100 mg dose revealed a significant decrease in the proportion of neutrophils and eosinophils and increase in monocytes, basophils, and γδ-T cells in the blood, which could be seen within the first to second hour post-drug treatment (Mustjoki et al., 2013). In addition, this study demonstrated enhanced cytotoxicity of NK cells (Mustjoki et al., 2013). Likewise, another *ex-vivo* study using white blood cells from healthy adults incubated with varying concentrations of dasatinib also showed a significant increase in γδ-T cells and NK cell proliferation (Wu et al., 2014).

We know much less about the effects of quercetin on innate immune cells; however, it has been shown to impact microglia due to its ability to pass through the blood-brain barrier (Han et al., 2021). In this study, treatment of murine microglial (BV2) cells with quercetin *in vitro* showed attenuation of inflammation (NFk-B activation) and reactive oxygen species production and promoted mitophagy in response to LPS. *In vivo,* quercetin also promoted mitophagy in response to LPS mitochondrial damage and microglial-mediated neurotoxicity in mice. In another model in which mouse dendritic cells were treated with LPS and quercetin *in vitro* for up to 24 h, quercetin diminished dendritic cell responses by reducing MHC class II, costimulatory molecules, and cytokine and chemokine secretion (Huang et al., 2010). This could indicate quercetin may have a protective effect in sepsis and neuroinflammation related to brain injury and inflammatory disorders that are associated with age such as stroke.

## Senolytic drugs and adaptive immune cells

The adaptive immune system plays a unique role in pathogenic responses. Unlike the innate immune system, which is dependent on PRRs to recognize PAMPs and DAMPs, adaptive immune cells recognize and distinguish self from foreign antigens and orchestrate appropriate immune pathways that are pathogen specific (Medzhitov and Janeway, 2000). Its key functions are highly specialized and tailored to the individual pathogen encountered. T cells and B cells, major components of the adaptive immune system, recognize and bind to specific antigens presented by antigen-presenting cells, such as macrophages and dendritic cells (Medzhitov and Janeway, 2000). This recognition leads to the activation and proliferation of antigen-specific lymphocytes, enabling a targeted immune response. Another vital function is the generation of immunological memory. Upon encountering a pathogen for the first time, certain lymphocytes differentiate into memory cells, which can recognize and mount a rapid and robust response upon subsequent exposure to the same pathogen (Pennock et al., 2013). This process forms the basis of immunization and provides long-lasting protection. Additionally, the adaptive immune system exhibits immunological tolerance to prevent immune reactions against the body’s own tissues (Pennock et al., 2013). Furthermore, the adaptive immune response involves the production of antibodies by B cells, which can neutralize pathogens or mark them for destruction by other immune cells (LeBien and Tedder, 2008). Overall, the adaptive immune system orchestrates highly specific and targeted immune responses, resulting in a diverse pathogen recognition repertoire and establishing long-term immunity. It is largely unknown if senolytic drugs will affect immunological memory, however studies assessing fisetin, dasatinib, and quercetin and adaptive immunity will be discussed here.

A study, described earlier, examining the effect of fisetin treatment in a murine model of allergic asthma regulation showed fisetin reduced the number of total lymphocytes and serum IgE, where abnormal elevation is characteristic of eosinophilic asthma (Huang et al., 2018). Comparably, another study examining allergic asthma in mice using ovalbumin sensitization, showed a significant reduction in the number of CD4 and CD8 T cells, and B cells in the lungs and bone marrow (Mitra et al., 2022). A significant decrease in serum ovalbumin specific IgE antibody levels with oral treatment of 50 nM fisetin (given 1 hour prior to ovalbumin exposures during sensitization period) was also observed (Mitra et al., 2022). In human B lymphoblastoid cells (Raji cells), fisetin induced apoptosis in a dose-dependent manner *in vitro* where cell viability was significantly decreased with 1 uM fisetin after 72 h, 3 uM fisetin after 48 h, and with 10 and 30 uM fisetin after just 24 h (Lim et al., 2015). Cell culture media supplementation with 10 uM fisetin resulted in less than 50% B cell viability when compared to control untreated cells after 72 h and likewise, 30uM fisetin resulted in ∼15% viability (Lim et al., 2015). This study also showed Raji cells treated *in vitro* with 10uM fisetin had impaired PI3-kinase activity (α, β, δ, γ isoforms) and 30uM fisetin inhibited mTOR pathway signaling and induced DNA damage (i.e., upregulation of γH2A.X) (Lim et al., 2015). In the context of immunity, cell number can only provide so much information; assessing differentiation and function are necessary to determine the overall consequences of fisetin on immunity.

Another study examined the immunosuppressive effects of fisetin in the context of CD4 T cell activation (Song et al., 2013). CD4 T cells from young mice treated with concanavalin A stimuli and fisetin (0-4 ug/mL) *in vitro* for 48 h suppressed overall proliferation in a dose-dependent manner, where 2-4 ug/mL showed significant reduction in proliferation compared to 0 or 1 ug/mL (Song et al., 2013). Interestingly, this was also shown with splenocytes *in vitro* where higher doses (8–16 ug/mL) resulted in a significant decrease in overall viability as compared to 0-4 ug/mL (Song et al., 2013). The ratio of CD4 to CD8 in T cells and production of CD4 T cell helper type I associated cytokines (i.e., IFN-γ, IL-2) was decreased by fisetin in a dose-dependent manner. It instead promoted a CD4 T cell helper type II cytokine profile (i.e., IL-4 and IL-6 secretion) indicating an alteration in subset differentiation (Song et al., 2013). These two studies reveal the immunosuppressive effects of fisetin in animal models, where lower doses may be effective at preventing disease progression, particularly in allergic asthma and diseases where immunosuppression is essential, however, higher doses of fisetin may have a significant immunosuppressive effect on adaptive immune cells. Fisetin also seems to have an impact on differentiation of CD4 T cells and reduced the overall proportion of CD4s when compared to CD8 T cells. This is important to bear in mind when considering the use of fisetin as a therapeutic intervention in disease. Fisetin treatment may be beneficial in allergic asthma or delayed hypersensitivity responses but may have adverse effects on immune cell function in the context of infectious disease where a robust and dynamic T and B cell responses are essential for mediating pathogen clearance and tissue recovery.

The senolytic capacity of fisetin was explored in a thorough 2018 study and included key insights into its effects on adaptive immune cells (Yousefzadeh et al., 2018). For instance, one experiment included progeroid mice fed standard chow *ad libitum* supplemented with or without 500 ppm (500 mg/kg) fisetin between 10 and 20 weeks of age (Yousefzadeh et al., 2018). These mice age more rapidly than wild-type mice and undergo accelerated aging making them a useful model in senescence research. Progeroid mice fed chow supplemented with fisetin had a significant reduction in mRNA gene expression of *p16*, *p21*, and other SASP markers (e.g., IL-6, TNF-α, IL-1β) in fat, spleen, liver, kidney, and peripheral CD3^+^ T cells measured at 20 weeks of age (Yousefzadeh et al., 2018). This was also demonstrated in wild-type mice when fed fisetin supplemented chow in the same manner between 85 and 120 weeks of age (∼20–30 months) (Yousefzadeh et al., 2018). Wild-type mice fed fisetin supplemented chow from 85 weeks through end of life, also had significant lifespan extension (Yousefzadeh et al., 2018). Taken together, fisetin can reduce some hallmarks of aging in whole tissues and T cells over a long exposure period and when administered later in life.

This study also leveraged the p16^INK−ATTAC^ mouse model, which contains a construct under the control of the p16 promoter allowing for identification and elimination of p16^Ink4a^ expressing cells (Baker et al., 2011; Yousefzadeh et al., 2018). The flag-tagged FKBP-Casp8 fusion protein of this construct was used to quantify p16^Ink4a^ expressing cells in adipose tissue of young, naturally aged untreated, and naturally aged fisetin-treated p16^INK−ATTAC^ mice (Yousefzadeh et al., 2018). Short term treatment of fisetin (100 mg/kg for 5 consecutive days by oral gavage) resulted in a significant decrease in the proportion of flagged p16^Ink4a^ expressing c-kit^+^ cells, CD4^+^ and CD8^+^ T cells, NK cells, and endothelial cells within adipose tissue when compared to aged untreated mice (Yousefzadeh et al., 2018). This analysis was performed 3 days following the final administered dose of fisetin, which has a rapid half-life of 0.09 h and a terminal half-life of 3.1 h (Touli et al., 2011). While many studies examine immune cell effects over the course of treatment with senolytic agents, this is one of few to include analysis of immune cells post-drug clearance. This suggests that fisetin can induce changes that may be long lasting in naturally aged mice, even after a short drug exposure period. These results might be an indication that p16-expressing T cells and NK cells contribute to the overall senescence tissue burden and can be controlled with senolytic intervention. Eliminating these cells may enhance overall immune cell surveillance and tissue health.

Dasatinib targets include Src family tyrosine kinases (SRKs) and BCR-ABL fusion proteins, among several others (Montero, et al., 2011). In T and B cells, SRKs are important in cell development, signaling, and activation (Gauld and Cambier, 2004; Palacios and Weiss, 2004; Stirnweiss, et al., 2013). BCR-ABL fusion proteins (most notably recognized as a genetic biomarker of leukemias) play a key role in pro-survival signaling and increasing resistance to apoptosis (Neshat, et al., 2000; Gu, et al., 2009). A study assessing the impact of dasatinib on peripheral blood CD3^+^ T cells isolated from healthy volunteers (baseline characteristics not specified), demonstrated its specificity for T cell receptors (TCR) (Schade et al., 2008). 10nM and 100 nM dasatinib *in vitro* impaired TCR signaling transduction and inhibited downstream ERK phosphorylation (profoundly) and AKT phosphorylation (minimally) despite TCR stimulation (TCR-stim) (Schade et al., 2008). Cell culture media supplemented with 10 ng/mL IL-2, however, resulted in phosphorylation of AKT in the presence of dasatinib, indicating that dasatinib inhibits TCR signaling independently of IL-2 pathway activation and enough IL-2 can bypass dasatinib inhibition of TCR to still activate T cells (Schade et al., 2008). TCR-stim of total peripheral blood mononuclear cells (PBMCs) *in vitro* in the presence of 10 nM dasatinib showed a reduction in cell-cell interactions and responsiveness, as evidenced by a lack of T cell-monocyte clustering (Schade et al., 2008).

T cells incubated for 20 h in the presence of 10 nM dasatinib plus TCR-stim did not undergo apoptosis, rather they persisted in culture and showed a complete inhibition of CD69 expression, a marker of T cell activation, when compared to untreated TCR-stimmed controls (Schade et al., 2008). This CD69 inhibition was completely restored, however, when the same cultures were treated the following day for 16 h with a non-TCR stimulating agent (e.g., phorbol-12-myristate-13-acetate, PMA) (Schade et al., 2008). T cell proliferation was also inhibited when incubated with 10 nM dasatinib and restored with the addition of IL-2 (Schade et al., 2008). This study demonstrated the low cytotoxicity (i.e., minimal cell death) of dasatinib in healthy T cells and the ability to still become activated if IL-2 or other non TCR-stimulating agents are present. It is unclear if this same phenomenon would be observed in senescent T cells treated with dasatinib, however, this study and others showing similar results in CD4 T cells, CD8 T cells, and NK cells (Fei et al., 2008; Weichsel et al., 2008; Fraser et al., 2009) led to further groundbreaking research to understand the impact of dasatinib on adaptive immune cells and in CAR-T cell therapy.

It has been shown dasatinib disrupts confirmational changes that are necessary for SRK lymphocyte-specific protein tyrosine kinase (Lck) mediated signaling through the TCR after stimulation (Stirnweiss, et al., 2013) and therefore inhibits phosphorylation of CD3 TCR associated protein kinase ZAP70 (Mestermann, et al., 2019). While inhibiting TCR signaling is not beneficial in all cases considering TCR is indispensable for antigen recognition and initiating other key T cell functions, the impact of dasatinib on TCR can be leveraged to modulate CAR-T cell therapy, particularly in the case of CAR-T cell toxicity. 100nM dasatinib treatment of activated CD4 and CD8 CAR-T cells *in vitro* paused cell lysis and inflammatory signaling (Mestermann, et al., 2019). Removal of dasatinib from cell culture media resulted in near immediate re-activation of CAR-T cell functions (Mestermann, et al., 2019). This study further supports the findings that dasatinib can modulate T cell responses and healthy T cells will maintain their ability to function if they receive signals to bypass TCR inhibition or after removal/elimination of the drug. More research is needed, however, to determine the long-term effects of dasatinib treatment *in vivo* on memory and antibody responses. A 2015 study examining murine and human B cell effects of dasatinib demonstrated inhibition of B cell receptor signaling, much like CD4 and CD8 T cells (Oksvold et al., 2015). Unlike T cells, however, where viability was largely unaffected by dasatinib treatment, B cells demonstrated a significant increase in apoptosis *in vitro* with reductions in pre-B cells from human bone marrow and mature-human B cell populations (Oksvold et al., 2015).

Another study investigated peripheral CD4 and CD8 T cell and B cell migration and phenotype in patients with chronic myeloid leukemia (CML) (Colom-Fernández et al., 2019). Analysis was performed with blood taken prior to first treatment (pre-intake) and 2 hours post treatment (post-intake) with 100 mg dasatinib orally (Colom-Fernández et al., 2019). Using a transwell assay, CD4 and CD8 T cells had a significant reduction in migration towards CCL19 and CCL21, powerful chemoattractants, in CML patients post-intake compared to pre-intake (Colom-Fernández et al., 2019). This coincided with reduction in CCR7, which recognizes CCL19 and CCL21, post-intake, which could mean dasatinib impairs CD4 and CD8 T cell responsiveness to migratory signals (Colom-Fernández et al., 2019). This study also demonstrated a shift in phenotype of CD4, CD8, and B cells. There was a significant reduction in the percentage of naïve CD4 and CD8 T cells with an increase in effector memory subsets post-intake as well as a significant increase in the percentage of memory B cells post-intake (Colom-Fernández et al., 2019).

Like fisetin, quercetin treatment over the course of ovalbumin sensitization and challenge in a mouse model of asthma has also been shown to modulate T cell responses and promote a more CD4 T helper type I cell profile (Park et al., 2009). In a 2009 study, quercetin was shown to induce apoptosis and cell membrane permeability in leukemic cell lines, but not in normal healthy PBMCs *in vitro* when media was supplemented with either 10, 50, or 100uM quercetin following stimulation with IL-2, anti-CD28 monoclonal antibody, and staphylococcal enterotoxin B (SEB) (Lugli et al., 2009). 50 and 100uM quercetin also efficiently inhibited lymphocyte proliferation *in vitro*, but did not affect viability, even with phytohemagglutinin stimulation (Lugli et al., 2009). This was independent of cell cycle phase (Lugli et al., 2009). Lastly, when CD3^+^ T cells incubated with IL-2, anti-CD28 monoclonal antibody, and SEB *in vitro* were treated with 50 or 100uM quercetin, there was a significant decrease in the expression of activation markers CD38 and CD95 (Lugli et al., 2009). This indicates quercetin also has the ability to inhibit T cell activation, proliferation, and unlike dasatinib, cannot be compensated for by the administration of IL-2.

Taken together, senolytics have many direct immunological advantages, especially in the treatment of cancer, autoimmunity, and hypersensitivity reactions; conditions where immunosuppression is necessary to controlling disease outcomes and pathophysiology. We have very little insight, however, as to how these drugs affect immune cell function long-term or in the development of immunological memory. Although these drugs may be beneficial where it is important to dampen immune responses and prevent immune cell induced tissue damage and cytotoxicity, they may otherwise have very detrimental consequences in the context of vaccination and infectious disease.

## Senolytics in age-related disease

Eliminating SCs has proven to be a highly effective way of mitigating and even reversing disease in pre-clinical models of aging (Baker et al., 2011; Saccon et al., 2021; Wang L et al., 2022a; Chandra et al., 2022). Transgenic mouse models of SC identification and elimination are a powerful tool in measuring senescence burden within tissues (Baker et al., 2011; Xu et al., 2018; Yousefzadeh et al., 2019; Wang B et al., 2021; Shimada-Takayama et al., 2022). Senolytics have been used as pharmacological interventions for selective elimination of SCs in both genetically and non-genetically modified animals. Additionally, many of the current transgenic models are solely reliant on the high expression of singular biomarkers of SCs, which do not capture the diversity of senescent populations. Senolytic drugs, however, can target SCs more broadly by not being limited to high expression of p16^INK4A^ or p21^Cip1^ for ablation. In this section we discuss the use of senolytics in models of aging and age-related diseases.

A 2018 study assessed the senolytic potency of fisetin in primary murine embryonic fibroblasts (MEFs) from Ercc1^−/−^ mice, which will prematurely become senescent *in vitro* under oxidative stress (i.e., exposure to atmospheric oxygen, 20% O_2_, for several passages) (Yousefzadeh et al., 2018). Fisetin was able to significantly reduce SA-β-gal expression *in vitro* in a dose-dependent manner, where doses between 1 and 20uM reduced the senescent cell number compared to DMSO-treated cultures (Yousefzadeh et al., 2018). Using progeroid mice carrying a p16-luciferase reporter transgene that were fed *ad libitum* with standard chow supplemented with 500 ppm (500 mg/kg) fisetin between 6–8 weeks of age and then again between 12–14 weeks of age, fisetin was demonstrated to significantly reduce p16-dependent luciferase expression compared to mice fed unsupplemented chow (Yousefzadeh et al., 2018). Interestingly, the level of luminescence was significantly decreased compared to control mice during the periods without chow supplementation of fisetin between weeks 8–12 and 14–16 weeks (Yousefzadeh et al., 2018). This model of accelerated aging demonstrates the longer term suppressive effects of prior treatment of fisetin *in vivo*.

In a mouse model of sporadic Alzheimer’s Disease and dementia, senescence-accelerated prone 8 (SAMP8) mice showed improved cognitive and behavioral performance and an increase in proteins related to synaptic function in hippocampal tissue with fisetin treatment (Currais et al., 2018). D + Q was also shown to extend lifespan, improve many aspects of physical function (e.g., walking speed, grip strength, and endurance), and reduce inflammation and SC burden in various tissues (e.g., adipose tissue, intestine, heart, and bone) in aged mice (Roos et al., 2016; Farr et al., 2017; Xu et al., 2018; Yousefzadeh et al., 2018; Zhou et al., 2021). Additionally, it reduced gut inflammation, SC burden, and altered microbiota of the intestines suggesting that D + Q could ameliorate microbiome changes with age (Saccon et al., 2021).

In humans, as mentioned earlier, fisetin and D + Q, have been used in a variety of clinical trials. Scarcely any, however, include phenotypic and/or functional analysis of immune cells as part of their outcome measures in adults 65+ years of age (Table 1). Despite the evaluation of SASP, general inflammation, and other outcome measures in senolytic trials in older adults, this is not always indicative of immune cell function. Therefore, we would like to highlight those studies focusing on cellular immune responses in particular, which is an area of senolytic aging research in much need of exploration. Out of the studies included in Table 1, only one (DASAHIVCURE, NCT05527418) is in infectious disease. Even further, there are currently no clinical trials including these outcome parameters in adults older than 65 (DASAHIVCURE age range for study inclusion is 18–65). Four of the studies focus specifically on CD3^+^ T cells and two on total peripheral blood mononuclear cells (PBMCs) for analysis. While CD3^+^ cells typically represent the majority of immune cell types in peripheral blood (roughly 70–90%) making them relatively easy to isolate in great abundance and study, it is important to understand the impact senolytics have on other immune cell types and the aged immune system as a whole. This is especially true as each cell type has specialized functions that work in concert together and that may be altered depending on the dose and type of senolytic used. Important differences in the function and distribution of subpopulations of immune cells may be overshadowed by an oversimplistic classification of T cells and total PBMCs. Our knowledge of the effects of senolytics on immune cells in older adults (aside from cancer studies) is extremely limited, however current research using alternative models exploring the effects of senolytics on immune responses to infectious disease is advancing.

**TABLE 1:** Current clinical trials with immune-cell related outcome measures in older adults 65+ years of age using senolytics.

Condition	Senolytic agent used	Age	Summary of immune cell-related outcome measures^a,b^	Study phase	Estimated study start & completion date	NCT number
Osteoarthritis	Fisetin	40–85	Senescent PBMC quantification using flow cytometry	Phase 1 Phase 2	May 2021− June 2025	NCT04815902
Interstitial lung disease in CVID	Fisetin	18+	Immunophenotyping to examine changes in peripheral T cells	Phase 2	Mar 2023− December 2024	NCT05593588
Frailty in survivors of childhood cancer	Fisetin Dasatinib & Quercetin	18+	p16INK4a mRNA expression in CD3^+^ cells	Phase 2	June 2022− July 2024	NCT04733534
Alzheimer’s disease	Dasatinib & Quercetin	65+	Measurement of senescent CD3^+^ cells expressing p16	Phase 1 Phase 2	May 2022− June 2023	NCT05422885 (STAMINA)
Alzheimer’s disease	Dasatinib & Quercetin	65+	Measurement of senescent CD3^+^ cells expressing p16	Phase 2	Dec 2021− January 2023	NCT04685590 (SToMP-AD)
Recent HIV infection	Dasatinib monotherapy	18–65	Phenotyping of senescence markers in PBLs (Beta-galactosidase, Bcl-2, Histone H2A, p16 and CD87)	Phase 2	Nov 2022− December 2024	NCT05527418 (DASAHIVCURE)

^a^ The clinicaltrials.gov search criteria used to identify studies were ‘dasatinib,’ ‘fisetin,’ ‘ABT263,’ ‘UBX0101,’ ‘UBX1325,’ ‘senescence,’ and ‘senolytic’ in older adults aged 65+. Only studies that listed senescence analysis in immune cell types as primary and/or secondary outcomes were included in this table. Primary and secondary outcomes examining SASP, factors or inflammation associated with senescence alone were not included.

^b^ All studies have proposed to examine immune cells from whole blood.

Abbreviations: CVID, common variable immunodeficiency; NCT, national clinical trial; PBL, peripheral blood leukocyte; PBMC, peripheral blood mononuclear cell.

## Senolytic effects on infectious disease

More research studies are examining senescence in immune cell types, but very few have utilized senolytic drug treatment as an approach to improving immune cell function in chronic or acute infectious disease. In light of the COVID-19 pandemic and underlying senescence-associated and immune-related pathology, senolytic treatment has become of particular interest as a means to mitigate symptoms and effects of infection that result in fatality or progression to long-COVID. A recent COVID study examined senescence in isolated PBMCs from patients who were previously hospitalized and undergoing pulmonary evaluation 3 months post-hospitalization. Interestingly, p16^INK4a^ expression in total PBMCs was significantly associated with COVID-related pulmonary pathology (Lekva et al., 2022). Although it has been demonstrated that viruses can themselves be inducers of senescence (viral induced senescence, VIS) (Lee et al., 2021), much less is known about clearance and immunological outcomes during and after infection. Another COVID study explored senescence and senolytic treatment in several models (Lee et al., 2021). Human nasopharyngeal, upper airway mucosa samples, and lung tissue from deceased patients who succumbed to COVID-19 infection revealed highly increased SC burden as compared to those never infected with COVID-19 (Lee et al., 2021).

Single-cell RNA sequencing of nasopharyngeal samples from those who succumbed to COVID-19 also revealed higher transcript levels of p16^INK4a^ in ciliated respiratory epithelial cells and macrophages (Lee et al., 2021). *In vitro*, primary human nasal epithelial cells genetically engineered to stably overexpress SARS-CoV-2 receptors (HNEpC-hACE2) were infected with SARS-CoV-2 to induce VIS (Lee et al., 2021). These cells were then incubated with either navitoclax, fisetin, or D + Q, all of which showed a significant reduction in viability of VIS cells indicating VIS sensitivity to senolytic drugs (Lee et al., 2021). The use of senolytic drugs D + Q or navitoclax also showed significant reduction in pathology, SASP, and macrophage and neutrophil infiltration into nasopharyngeal and lung tissues of SARS-CoV-2 infected golden hamsters and dwarf hamsters (Lee et al., 2021). Finally, a third COVID study examined the response to infection in aged mice with and without fisetin treatment demonstrating a decrease in inflammation, SASP, p16^INK4a^ and p21^Cip1^ expression, as well as a significant improvement in survival and antigen-specific antibody titers (Camell et al., 2021).

Other work has examined the connections between SCs in aging and immunity during influenza (flu) infection. In a recent published study, differentiation of CD4 T cell helper subsets in aged flu infected mice demonstrated marked skewing to a regulatory T cell (Treg) phenotype when compared to young infected mice (Lorenzo et al., 2022). This is significant since the presence of increased levels of Tregs has the potential to negatively impact the adaptive immune response to flu infection (Lorenzo et al., 2022). This skewing was found to be the result of increased levels of TGF-β in the aged lungs and could be halted by the administration of TGF-β neutralizing antibodies. This point is important since TGF-β can be generated by SCs and is often found as a component of SASP (Hoare et al., 2016; Borodkina et al., 2018). Additionally, treatment of aged mice with D + Q followed by flu infection resulted in a significant decrease in Tregs and higher proportion of Th2 CD4 helper cells, which can aid in the resolution of infection. Due to the 4- and 11-hour half-lives of D + Q, respectively (Graefe et al., 2001; Christopher et al., 2008), treatment was administered over the course of 3 weeks with a 5 days resting period to ensure clearance of the drug to eliminate the possibility of off target drug effects, as opposed to examining the effect of SC elimination on CD4 T cells prior to infection. Mice treated with D + Q also resulted in significantly lower levels of TGF-β in the lungs during flu infection of aged mice, which has also been shown in D + Q studies using aged hamsters infected with COVID-19 (Blanco-Melo et al., 2020; Lorenzo et al., 2022). These results suggest a role for SCs and the senescent environment in the differentiation of immune cells. In fact, when young CD4 T cells were transferred into aged flu infected mice, they also demonstrated significantly enhanced differentiation to a Treg phenotype, which was abrogated by prior D + Q treatment as well (Lorenzo et al., 2022). While these results are encouraging, closer examination of the response to flu infection is needed to understand the impact on response to infection with senolytic treatments.

## Concluding remarks and considerations

**FIGURE 2:**
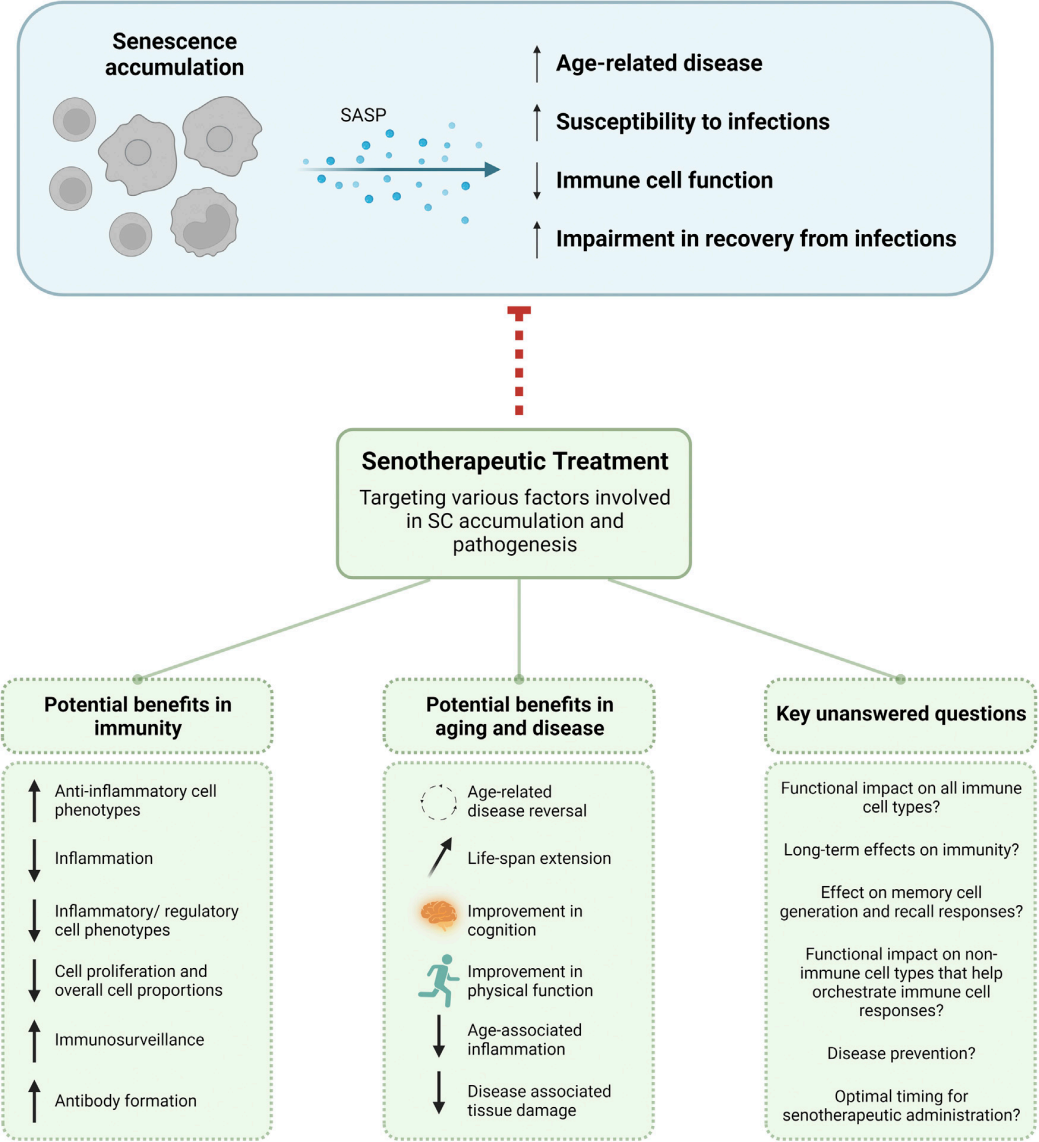
Potential benefits of senotherapeutic use and key unanswered questions. As SCs accumulate they secrete SASP reinforcing their own senescence phenotype and causing senescence in neighboring cells. This can lead to overall age-related disease, susceptibility to infections, immune cell function, and impairment in recovery from infections. With the use of senotherapeutics to target different facets of SC phenotypic and functional manifestations, there are potential benefits for use in immunity and aging. Care must be taken when using senotherapeutics in older adults to ensure the immunosuppression seen in many senescence models would not disrupt or inhibit proper immune responses and control of infections. There are also many unanswered questions as to the functional and long-term immunological effects. SASP, senescence-associated secretory phenotype; SC, senescent cell.

Notable and promising outcomes have been demonstrated with treatment of senolytics in diverse model systems, albeit not all these systems used these drugs specifically for their senolytic potential. Regardless, we are uncovering many consequences of senolytic treatment, especially as it relates to immunity, aging, and disease (Figure 2). In this review we focused on choice senolytics that are currently in clinical trials in older adults and include analysis of immune cells. There are, however, a rapidly increasing number of alternative strategies to target various senescence initiation and pro-survival pathways, immune evasion mechanisms, SASP, and cellular biomarkers to eliminate SCs. It is clear SCs can contribute to disease pathogenesis, but it is critical to consider the functional role senescence has in other cell processes like tissue regeneration and recovery from infection. Older adults with advanced diabetes who are at substantial risk for infections, skin lesions, and amputations (Lin et al., 2020; Salari et al., 2020), for example, may have serious long-term complications in tissue healing and recovery without SCs, as it has long been appreciated that SCs are crucial in wound healing (Demaria et al., 2014). More recently, a refined transgenic mouse model allowing for selective deletion and highly sensitive reporting of p16^INK4a^ expressing cells revealed senescent fibroblasts form a unique niche amidst stem cell populations in the lung that promote epithelial cell regeneration and healing of lung tissue following injury (Reyes et al., 2022). If true of human lung tissue, this may play a role in the recovery from lung infections such as COVID. It may therefore be beneficial to intervene with senotherapeutics at the peak of infection or post-pathogen clearance with the hopes that senescent lung fibroblasts will initiate epithelial cell repair and tissue healing, but not allow for the extensive accumulation of SCs, which may then have a negative effect and contribute to long-COVID complications. If treated too early, it is plausible that older adults with flu or COVID infection may have difficulty in initial stages of lung tissue repair without SCs. It would of course be necessary to determine if certain treatment regimens should be given differentially based on comorbidities, age, frailty status, *etc.* Other costs and benefits of using senotherapeutics in human disease have been previously reviewed (Raffaele and Vinciguerra, 2022).

## Future perspectives

There is a wide area of opportunity to fill gaps in our knowledge with regards to senotherapeutic effects on immune cells. In the context of immunity and aging, it will be important to determine if senolytics can overcome the age-related cell intrinsic and extrinsic declines in the immune system. Many of the immunosuppressive effects of senolytics discussed here from both *in vitro* and *in vivo* studies have collectively demonstrated an impairment in immune cell function, activation, and proliferation in a dose-dependent manner. At the same time, higher doses of senolytic drugs have also been shown to decrease inflammation. It would therefore be important to determine if inflammation can be reduced with senolytics while still preserving long-term immune cell function in cases where immunosuppression is not advantageous. It is also critical to understand how senolytic treatment affects tissue healing and resolution of infectious disease with age as well as the impact on memory cell generation. With the D + Q associated abrogation seen in TGF-β after flu infection in mice (Lorenzo et al., 2022), which is required for proper CD4 and CD8 memory formation, it is possible that senolytics could enhance an acute immune response, but considerably dampen immunological recall and/or vaccination responses.

Other outstanding questions include: 1) Do senotherapeutics directly affect immune cells? While some studies have described direct and indirect effects on immune cells (discussed earlier), this seems to be dependent on senolytic type, treatment regimen, and model system and we do not yet understand the long-term implications senotherapeutics have. 2) Do senotherapeutics differentially affect immune cell types? It would be very advantageous to understand if certain senotherapeutics can be used for targeted enhancement of particular subsets in models of infection and disease with aging or if different therapies or treatments should be used simultaneously for more broad affects. Comprehensive head-to-head comparisons of different senolytics assessing functions of immune cell types are also needed. 3) Is there an association between SC burden and general immune cell decline that could be an indicator of when to administer senotherapeutics in older adults? It would be interesting to essentially develop an alternative aging clock (Sayed et al., 2021) including parameters related to SC accrual, deficits in immune cell subtypes, comorbidities, susceptibility to disease, and predict optimal treatment strategies and regimens. 4) Do they alter structural integrity of tissues that may impact immune function, migration, and cross-talk? Loss of lymph node and splenic architecture have been reported to contribute to functional immune decline with age (Masters et al., 2018; Masters et al., 2019) and it is possible fibroblastic SCs in these tissues have negative spatiotemporal affects on immune cells. 5) Do senotherapeutics induce long-term enhancement of immune surveillance? While we have seen this in acute models as described earlier, is this maintained over time?

Although we do not have enough data to support administration of senolytic drugs at concrete times over the course of one’s lifespan, it has been proposed to intermittently treat over a set short period of time (e.g., several treatments per day over the course of a few days, one treatment per day over the course of several weeks, or weekly treatments over the course of several months). Much more research will be needed to determine if the use of a particular treatment regimen and dosage should be dependent on disease state or other clinical, biological, or environmental factors. It is also important to determine how long the effects of senolytic treatments last and how these treatments may differentially impact susceptibility to and recovery from disease. Despite potential challenges, we have many more advanced tools and methods of studying SCs and senolytics, which enable researchers to investigate these more nuanced questions and ensure long-term safety as senolytics are used on- and off-label in the clinic.
